# Rhinoplastic Operation

**Published:** 1872-12

**Authors:** H. H. Toland


### ARTICLE VIII.

### *Rhinoplastic Operation.*

BY H. H. TOLAND, M. D.

John Krimmel was admitted into the County Hospital, September 8th, 1870, in the condition represented.

There was no evidence that the difficulty resulted from syphilis, and being delicate, the ulceration by which the nasal organ was destroyed was regarded as scrofulous. Consequently a generous diet, combined with tonics, was prescribed until his general health was considerably improved, which treatment would have been continued longer before the operation was performed if he had not presented evidences of mental derangement—which I attributed to the distress produced by the loss of so important and prominent an organ as the nose.

The skin covering the frontal bone was exceedingly thin, which rendered the result much more uncertain.

The operation was performed in the usual manner. The integument taken from the forehead was secured by the interrupted silver sutures, which were allowed to remain ten days, so that should adhesion by the first intention fail to take place, it would occur by granulation. The wound on the forehead was closed, and the edges held in opposition, except the superior portion by the interrupted silver sutures. At the expiration of seven days, instead of dividing the attachment of the transplanted portion of integument, it was adjusted to what remained of the superior part of the nose, adhesion by the first intention took place, and the circulation was thereby greatly increased. After this attachment was formed, the transplanted portion being still thin and shapeless, I determined to give it additional support by connecting the sides with flaps raised from the face, which, when accomplished, improved the prospect of success very materially. The nose was gradually brought into a proper shape by the use of sections of an elastic catheter covered with oiled lint and secured by adhesion plaster. If the ordinary operation had been relied upon, it would have been a failure, and the opening would have been simply covered with a portion of healthy integument, entirely unlike the original organ.

In this operation the attachment or pedicle should not be stretched so as to produce strangulation. At the expiration of an hour after the incisions have all been made, the blood should be carefully removed from the entire surface of the parts designed to be approximated, and they should be held in contact by the interrupted silver suture, with occasionally a fine silk ligature, to effect a more perfect adaptation of the parts. The latter should be removed on the third or fourth day, and the former not for eight or ten days, unless they cease to be useful. To prevent a failure in such cases drainage must be secured, which can be accomplished by the application of simple cerate, the water dressing, or both. Sometimes it is necessary to apply the simple cerate over the wound, and the warm water dressing externally, to maintain the heat and increase the circulation of the part. Should the escape of the bloody serum, which is found on all wounded surfaces, be prevented, it accumulates between the surfaces, which renders union by the first intention impossible.

When the nose is only partially destroyed, the portion of skin necessary to supply the deficiency may be taken either from the side of the face or forehead, as may be most convenient, and the pattern being cut of pasteboard, leather or blotting paper, should be placed upon the part selected, and the size and shape marked with an ordinary pen and ink, the trace of which, when dry, can be easily followed. Care should be taken to avoid both the cellular tissue and periosteum, for the removal of the latter would cause an enfoliation of the denuded bone, and prove a source of annoyance for several months. In cases of deformity, resulting from destruction of the eyelids, either partial or complete, the side of the forehead or face will be found equally convenient, and in extropium of either or both lids, the same locality may be selected, and in consequence of the result, it is one of the most important plastic operations that has ever been performed, and one that never fails when properly executed. Sometimes the transplanted portion remains thickened for a considerable time; in such cases the projecting portion can be removed and the wound healed by the ordinary treatment.— *Western Lancet.*

